# *Escherichia albertii* in Wild and Domestic Birds

**DOI:** 10.3201/eid1604.090695

**Published:** 2010-04

**Authors:** J. Lindsay Oaks, Thomas E. Besser, Seth T. Walk, David M. Gordon, Kimberlee B. Beckmen, Kathy A. Burek, Gary J. Haldorson, Dan S. Bradway, Lindsey Ouellette, Fred R. Rurangirwa, Margaret A. Davis, Greg Dobbin, Thomas S. Whittam

**Affiliations:** Washington State University, Pullman, Washington, USA (J.L. Oaks, T.E. Besser, G.J. Haldorson, D.S. Bradway, F.R. Rurangirwa, M.A. Davis); University of Michigan Health System, Ann Arbor, Michigan, USA (S.T. Walk); The Australian National University, Canberra, Australian Capital Territory, Australia (D.M. Gordon); Alaska Department of Fish and Game, Fairbanks, Alaska, USA (K.B. Beckmen); Alaska Veterinary Pathology Services, Eagle River, Alaska, USA (K.A. Burek); Michigan State University, East Lansing, Michigan, USA (L. Ouellette, T.S. Whittam); University of Prince Edward Island, Charlottetown, Prince Edward Island, Canada (G. Dobbin); 1 Deceased.

**Keywords:** Avian, enteritis, *Escherichia*, pathology, bacterial toxins, veterinary, intimin, cytolethal distending toxin, bacteria, research

## Abstract

*Escherichia albertii* has been associated with diarrhea in humans but not with disease or infection in animals. However, in December 2004, *E. albertii* was found, by biochemical and genetic methods, to be the probable cause of death for redpoll finches (*Carduelis flammea*) in Alaska. Subsequent investigation found this organism in dead and subclinically infected birds of other species from North America and Australia. Isolates from dead finches in Scotland, previously identified as *Escherichia coli* O86:K61, also were shown to be *E. albertii*. Similar to the isolates from humans, *E. albertii* isolates from birds possessed intimin (*eae*) and cytolethal distending toxin (*cdtB*) genes but lacked Shiga toxin (s*tx*) genes. Genetic analysis of *eae* and *cdtB* sequences, multilocus sequence typing, and pulsed-field gel electrophoresis patterns showed that the *E. albertii* strains from birds are heterogeneous but similar to isolates that cause disease in humans.

## Abstract 2

The isolates were similar to those that cause disease in humans.

In late December 2004, deaths of common redpoll finches (*Carduelis flammea*) were reported around the city of Fairbanks, Alaska, USA, coincident with a prolonged period of extreme cold (below –40°F). The final reported death occurred on February 24. At the beginning of the outbreak, the local at-risk population was estimated to be ≈8,000 redpoll finches, a historic high for the area. Although ≈100 deaths were documented, the actual number is assumed to be considerably higher.

Outbreaks of disease in wild finches (family Fringillidae) have been associated with *Salmonella enterica* subsp. *enterica* serotype Typhimurium, *Mycoplasma gallisepticum*, poxvirus, and *Escherichia coli* (*1*–*6*). Diagnostic investigation into the Alaska outbreak identified *Escherichia albertii* as the probable cause of death and as a new pathogen for birds. *E. albertii* had been identified as an enteric pathogen of humans in Asia (*7*) and, more recently, in Africa and North America (T.S. Whittam and H. Steinsland, unpub. data), but to our knowledge, until this outbreak its presence in animals had not been observed.

We describe the identification and characterization of *E. albertii* from birds in North America, Europe, and Australia. We show that bacterial isolates from dead finches in Scotland, previously identified as *E. coli* O86:K61, were actually *E. albertii*. The genetic diversity of 2 virulence loci (intimin and cytolethal distending toxin) for the bird isolates was compared with characterized human pathotypes. We also determined genetic relatedness among isolates from birds and humans by multilocus sequence typing (MLST) and clonality of multiple isolates from dead or clinically healthy birds by pulsed-field gel electrophoresis (PFGE).

## Methods

### Bird Isolate Collection

In the United States during 2004–2005, dead redpoll finches from Alaska were submitted to the Alaska Department of Fish and Game. Three clinically healthy redpoll finches were trapped near the outbreak site. Standard necropsies included gross examination and collection of tissues into 10% neutral buffered formalin for histopathologic examination. Fresh tissues were frozen for microbiologic assays. Two other dead birds, a captive adult gyrfalcon (*Falco rusticulos*) from Idaho and a chicken (*Gallus gallus*) from Washington, were submitted for diagnosis to the Washington Animal Disease Diagnostic Laboratory in Pullman, Washington, USA.

In Canada in 2005, isolates were obtained from feces of clinically healthy redpolls and pine siskins (*Carduelis pinus*) trapped on Prince Edward Island. A total of 158 finches were sampled and included redpolls, pine siskins, and purple finches (*Carpodacus purpureus*).

In Australia in 2001–2002, isolates from birds were obtained from feces of clinically healthy domestic and trapped wild birds (*8*). Domestic birds included 9 chickens (*G. gallus*), 4 geese (*Anser anser domesticus*), 3 ducks (*Anas platyrhynchos domesticus*), and 1 guinea fowl (family Numididae). Wild birds totaled 634 birds representing 112 species.

In Scotland, isolates obtained during 1998–2000 from dead birds—Eurasian siskins (*Carduelis spinus*), greenfinches (*Carduelis chloris*), and chaffinches (*Fringilla coelebs*)—previously identified as *E. coli* O86:K61 (*4*), were obtained from M.J. Woodward (Veterinary Laboratories Agency Weybridge, Inverness, UK). Information about all isolates is summarized in Table 1.

**Table 1:** *Escherichia albertii* isolates and hosts information

Isolate	Origin (state)*	Host species of origin	Clinical status	Year of isolation	Reference
1568-05-27C†	USA (AK)	Redpoll finch (*Carduelis flammea*)	Dead	2005	This study
1568-05-27D†	USA (AK)	Redpoll finch (*C. flammea*)	Dead	2005	This study
1615-05-A†	USA (AK)	Redpoll finch (*C. flammea*)	Dead	2005	This study
1615-05-B†	USA (AK)	Redpoll finch (*C. flammea*)	Dead	2005	This study
1297-05-19†‡§¶	USA (AK)	Redpoll finch (*C. flammea*)	Dead	2005	This study
7991-07†‡§¶	USA (WA)	Chicken (*Gallus gallus*)	Dead	2007	This study
12055-07†‡§¶	USA (ID)	Gyrfalcon (*Falco rusticulos*)	Dead	2007	This study
5419-05-R†‡§¶	Canada	Redpoll finch (*C. flammea*)	Healthy	2005	This study
5419-05-S†‡	Canada	Pine siskin (*Carduelis pinus*)	Healthy	2005	This study
EC370-98†‡§¶	Scotland	Finch spp. (*Carduelis* spp.)	Dead	1998	(*9*)
EC558-00†	Scotland	Eurasian siskin (*Carduelis spinus*)	Dead	2000	(*9*)
EC744-99†	Scotland	Greenfinch (*Carduelis chloris*)	Dead	1999	(*9*)
EC746-99‡	Scotland	Eurasian siskin (*C. spinus*)	Dead	1999	(*9*)
EC748-99†	Scotland	Greenfinch (*C. chloris*)	Dead	1999	(*9*)
B090†‡¶	Australia	Magpie (*Gymnorhina tibicen*)	Healthy	2001	This study
B101†‡§¶	Australia	Magpie(*G. tibicen*)	Healthy	2001	This study
B156†‡§¶	Australia	Magpie (*G. tibicen*)	Healthy	2001	This study
B249†‡¶	Australia	Magpie (*G. tibicen*)	Healthy	2001	This study
B198†‡§¶	Australia	Honeyeater (*Melithreptus brevirostris*)	Healthy	2002	This study
B992†‡§¶	Australia	Wren (*Malurus cyaneus*)	Healthy	2001	This study
B1086†‡§¶	Australia	Fantail (*Rhipidura fulginosa*)	Healthy	2002	This study
B1068†‡§¶	Australia	Chicken (*G. gallus*)	Healthy	2002	This study
B1074†‡§¶	Australia	Chicken (*G. gallus*)	Healthy	2002	This study
616‡	No data	Human	Diarrhea	No data	(*10*)
3103-99‡	USA (IL)	Human	Diarrhea	No data	(*10*)
C-425‡§	No data	Human	Diarrhea	No data	(*10*)
106A5‡§	Guinea-Bissau	Human	Healthy	1997	(*11*)
9194†‡	Bangladesh	Human	Diarrhea	1990	(*10*)
19982‡§¶	Bangladesh	Human	Diarrhea	1990	(*10*)
79D4†‡	Guinea-Bissau	Human	Healthy	1997	(*11*)
97F8‡	Guinea-Bissau	Human	Healthy	1997	(*11*)
M2005000616 #8‡	USA (MN)	Human	Diarrhea	No data	T.S. Whittam, unpub data
I2005002880 #36‡	USA (MN)	Human	Diarrhea	No data	T.S. Whittam, unpub data
K-694¶	Bangladesh	Human	Diarrhea	No data	(*10*)
K-1‡§¶	Bangladesh	Human	Diarrhea	No data	(*10*)

*AK, Alaska; WA, Washington; ID, Idaho; IL, Illinois; MN, Minnesota. †Included in pulsed-field gel electrophoresis analysis. ‡Included in multilocus sequence typing analysis. §Included in *eae* allele analysis. ¶Included in *cdtB* allele analysis.

### Isolation and Identification of Bacteria

To detect Enterobacteriaceae, we inoculated tissues, intestinal contents, or feces onto MacConkey agar and incubated the plates at 35°C. Isolated colonies were characterized by fermentation of lactose and glucose, production of oxidase, and production of indole from tryptophan. Additional biochemical characterization was performed by using a commercial kit (API 20E; bioMérieux, Hazelwood, MO, USA). *E. coli* serotyping was performed by the Gastroenteric Disease Center (Wiley Laboratory, Pennsylvania State University, University Park, PA, USA).

Genetic identification was based on 16S rRNA gene sequencing and/or PCR to detect housekeeping gene polymorphisms unique for the *E. albertii*/*Shigella* *boydii* lineage. 16S rRNA analysis was performed on 1 isolate from Alaska by sequencing >1,400 nt of the 16S rRNA gene (*12*). The amplicon was cloned into the pCR2.1 sequencing vector (TOPO TA Cloning Kit; Invitrogen, Carlsbad, CA, USA) and sequenced bidirectionally by automated dideoxy DNA methods. Partial 16S rRNA sequences of ≈500 bp of the 5′ end, including the V1, V2, and V3 variable regions (*13*), were determined for other isolates with the same primers, after which direct dideoxy sequencing was performed. A sequence similarity search was performed by searching the GenBank database with BLASTN.2.2.3 (*14*), and sequences were aligned with ClustalW2 (www.ebi.ac.uk/Tools/clustalw2/index.html). PCR was used to detect *E. albertii* lineage–specific genetic polymorphisms in the housekeeping genes *lysP* and *mdh* (*10*). As a positive control, PCR for the gene *clpX*, which is conserved in *E. coli*, *Shigella*, and the *E. albertii*/*S. boydii* lineage, was performed as described (*10*), except a corrected primer sequence for *clpX*_28 (5′-TGG CGT CGA GTT GGG CA-3′) (T.S. Whittam, unpub. data) was used. The negative control for the *lysP* and *mdh* PCR was *E. coli* strain DH10b.

### Virulence Gene PCR and Sequence Analysis

PCR was used to test isolates for virulence genes found in Enterobacteriaceae—the central conserved region of intimin (*eae*, the attaching-and-effacing ligand), heat-stable enterotoxin (*sta*), and Shiga toxins (*stx1* and *stx2*)—as described (*15*). Positive controls were *E. coli* strains S2 (for *sta*) and S14 (for *stx1*, *stx2*, and *eae*) from the Pennsylvania State University *E. coli* Reference Center. A multiplex PCR protocol that amplified the consensus portion of the B subunit of the cytolethal distending toxin gene (*cdtB*) as described by Toth (*16*) was modified by using each of the primer pairs (s1/as1 and s2/as2) individually to screen for *cdtB* in all isolates.

For sequencing *eae*, PCR primers that amplified ≈800 nt of the variable 3′ end of the *eae* gene were used as described (*9*). Because these primers did not work for all bird isolates, additional primer sequences were either taken from other studies or designed for this study (Tables 2 and 3). Sequence analysis was based on ≈726 nt in the 3′ variable region of *eae*, which corresponded to amino acids 33–275 within the C-terminal 280 aa of intimin (Int280). Nucleotide sequences were determined for each of the *cdtB* products obtained by using the s1/as1 (403 bp) and s2/as2 (411 bp) primer pairs (*16*). Predicted amino acid sequences for *eae* and *cdtB* were aligned with reference alleles by using the ClustalW method and MegAlign software (DNASTAR, Madison, WI, USA). Neighbor-joining dendrograms were constructed by using MEGA version 4 (*18*) with the p-distance metric and pairwise gap deletion.

**Table 2:** Primers used for amplification and sequencing of *eae* gene of *Escherichia albertii*

Primer	Sequence, 5′ → 3′	Position (GenBank accession no.)	Reference
Intimin γ F	CGTTGAAGTCGAGTACGCCA	1867–1887 (AF081185)	(*9*)
Intimin γ R	TTCTACACAAACCGCATAGA	2782–2803 (AF081185)	(*9*)
EaeA-F	CAAACCAAGGCCAGCATTAC	1963–1982 (AF081185)	This study
EaeA-R outer	CCCCAAGAGAGAGGGTTCTT	2743–2763 (AF081185)	This study
EaeA-R inner	ACTTGATACCCCAGACCTTCA	2703–2725 (AF081185)	This study
EscD-R1	GTATCAACATCTCCCGCCA	27918–27937 (AF022236)	(*17*)
Intimin-R2	CAGAATATTAAACAAGCGCAGTTG	3103–3126 (FJ609833)	This study
EaeA F06s	GTAACGGACTTTACGGCTGATA	1803–1824 (FJ609833)	(*10*)
Intimin B101 int R	TGACCATATTGCAACCA	2460–2476 (FJ609833)	This study
Intimin B156 int R	TGACCATATCGCAACCA	2459–2475 (FJ609822)	This study

**Table 3:** Primer sets used for amplification and sequencing of *eae* gene of *Escherichia albertii* in specific isolates

Isolate	PCR amplification	Sequencing
1297–05–19	Intimin γ F, intimin γ R	Intimin γ F, intimin γ R
7991–07	EaeA-F, EaeA-R outer	EaeA-F, EaeA-R inner
12055–07	Intimin γ F, intimin γ R	Intimin γ F, intimin γ R
5419–05-R	Intimin γ F, intimin γ R	Intimin γ F, intimin γ R
EC370–98	Intimin γ F, intimin γ R	Intimin γ F, intimin γ R
EC746–99	Intimin γ F, intimin γ R	Intimin γ F, intimin γ R
B101	Intimin γ F, intimin γ R	Intimin γ F, intimin γ R
B156	EaeA-F, Intimin-R2	EaeA-F, intimin-R2, intimin B156 int R
B198	EaeA-F, intimin-R2	EaeA-F, intimin-R2, intimin B156 int R
B992	EaeA F06s, EscD-R1	EaeA F06s, EscD-R1, EaeA-F, intimin B101 int R
B1086	EaeA-F, intimin γ R	EaeA-F, intimin γ R, EaeA-R outer
B1068	EaeA F06s, EscD-R1	EaeA F06s, EscD-R1, EaeA-F
B1074	EaeA F06s, EscD-R1	EaeA F06s, EscD-R1, EaeA-F

### MLST

MLST was performed on 26 isolates of *E. albertii* (Table 1) as described (*10*), with slight modification. Briefly, partial gene sequences for 6 conserved housekeeping loci (*aspC*, *clpX*, *fadD*, *icdA*, *lysP*, and *mdh*) were obtained by PCR and direct sequenced by automated dideoxy sequencing. Raw sequences were aligned by using Seqman Pro software (DNASTAR). Sequences for 11 *E. albertii* isolates from humans and 6 common *E. coli* pathotypes were obtained from www.shigatox.net. *E. coli* was used as an outgroup; strains used were enterohemorrhagic *E. coli* strain EDL933, *Shigella flexneri* strain 2747-71, enteroaggregative *E. coli* strain 042, enteropathogenic *E. coli* strain e2348/69, uropathogenic *E. coli* strain CFT073, and *E. coli* K-12. A neighbor-joining dendrogram was based on the concatenated nucleotide sequence and the maximum composite likelihood model by using MEGA version 4 (*18*). Details of the MLST procedure, including allelic typing and sequence type assignment methods, can be found at www.shigatox.net.

**Figure 1:**
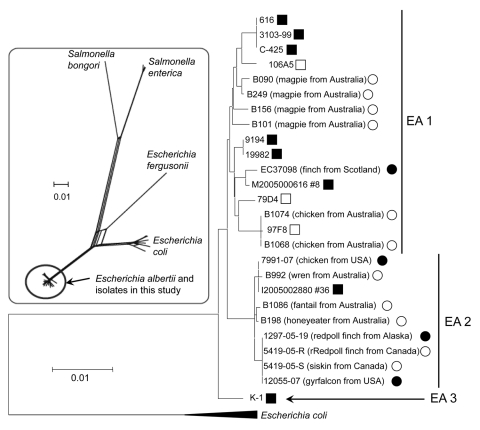
Neighbor-joining dendrogram of bird and human isolates, based on nucleotide variation at 6 conserved housekeeping loci in the *Escherichia albertii* genome (multilocus sequence typing), supporting identification of all isolates as *E. albertii*. Three distinct clades are designated EA 1–EA 3. No clustering of isolates is apparent on the basis of their host type, geographic origin, or association with disease. Inset is a SplitsTree phylogenetic network of the genus *Escherichia*, showing the *E. albertii*/*Shigella boydii* serotype 7/13 lineage with respect to the other named species. *S. bongori*, *Salmonella bongori*; *S. enterica*, *Salmonella enterica*. Scale bars indicate genetic distance.

An overall phylogenetic representation of the genus *Escherichia* was generated by combining nucleotide sequence data from GenBank for *Escherichia fergusonii* with outgroup strains *Salmonella bongori*, *S. enterica* subsp. *enterica* serotype Typhi, and *S. enterica* subsp. *enterica* serotype Typhimurium. A neighbor-net network analysis was generated by using SplitsTree 4 software (*19*) (Figure 1, inset).

### PFGE

*E. albertii* isolates were compared by using a standard PFGE method (*20*) with minor modifications. Briefly, fragments of *Xba*I-digested bacterial DNA were separated in 1% agarose gel by using a CHEF-DR III PFGE apparatus (Bio-Rad, Hercules, CA, USA); pulse times were ramped from 2.2 to 54.2 seconds over 19 hours. Digital gel images were analyzed with Bionumerics software (Applied Maths, Sint-Martens-Latem, Belgium) by using the unweighted pair group method with arithmetic mean algorithm for cluster analysis of Dice similarity coefficients with a position tolerance of 2%.

### Nucleotide Sequence Accession Numbers

Nucleotide sequences from this study were deposited in GenBank. Their accession numbers are EU926632–EU926649 and GQ140242–GQ140261.

## Results

### Pathologic Findings

Redpoll finches from the Alaska outbreak were typically found dead without obvious signs of disease. All those evaluated had adequate pectoral muscle mass, suggestive of acute death. Some had green fecal material pasted around their cloacae, suggestive of diarrhea. Gross lesions were inconsistent, but a few birds had darkened intestines distended with excessive yellow to green digesta. Histologic lesions were also inconsistent, but when present they were consistent with acute, severe, fibrinous, and necrotizing proventriculitis; multifocal heterophilic enteritis; and small-crypt abscessation. For some, gram-negative bacilli in large numbers were observed within the intestinal lumens. Attachment of bacteria to intestinal epithelial cells was not observed, although autolysis precluded assessment of the epithelium and ultrastructural studies to detect attaching-and-effacing lesions. No lesions consistent with septicemia were observed in any affected redpolls.

The affected gyrfalcon had appeared clinically healthy until found dead. Histologic examination failed to demonstrate enteric lesions, although evidence of septicemia was found. The chicken from Washington died after ≈1 week of illness, during which it appeared depressed and anorexic. Histopathologic examination showed severe, diffuse, necrotizing typhlitis; mild to moderate enterocolitis; and septicemia.

### Bacteria Isolated

Bacterial cultures were performed for 8 dead redpolls from Alaska and 3 healthy redpolls trapped in the same area. Large numbers of non–lactose-fermenting gram-negative rods were isolated from the intestines and tissues of 5 of the dead redpolls but from none of the 3 healthy redpolls. Similar organisms were also isolated in large numbers from the tissues of the gyrfalcon and intestines of the chicken. In the healthy birds trapped on Prince Edward Island, non–lactose-fermenting bacteria were isolated from the feces of 11 (12%) of 95 siskins and 4 (12%) of 33 redpolls but from none of 30 samples from purple finches. From the healthy birds trapped in Australia, non–lactose-fermenting bacteria were isolated from 4 (18%) of 22 magpies (*Gymnorhina tibicen*), 1 (10%) of 10 honeyeaters (*Melithreptus brevirostris*), 1 (3%) of 38 wrens (*Malurus cyaneus*), 1 (7%) of 15 fantails (*Rhipidura fulginosa*), and 2 (22%) of 9 chickens.

The isolates were oxidase negative; fermented glucose but not lactose, sucrose, or xylose; produced indole from tryptophan; and were nonmotile at 35°C. Further biochemical characterization with the API 20E panel indicated that the isolates produced lysine decarboxylase and ornithine decarboxylase and fermented d-glucose, d-mannitol, and l-arabinose. The isolates did not utilize citrate; did not produce arginine decarboxylase, hydrogen sulfide, urease, tryptophan deaminase, acetoin, or gelatinase; and did not ferment inositol, l-rhamnose, d-sucrose, d-melibiose, or amygdalin. All isolates except that from the fantail from Australia (B1086) used β-galactosidase. Fermentation of d-sorbitol varied; it was not fermented by the isolates from finches from Alaska, Canada, and Scotland or the gyrfalcon or by 3 of the 9 isolates from birds in Australia (B1068, B1074, and B1086). API 20E testing identified the isolates that used β-galactosidase and fermented d-sorbitol as code 5144102 and weakly (43%) identified them as *E. coli*. Identical API 20E profiles were reported for the isolates identified as *E. coli* from the dead finches from Scotland (*4*). Although the isolates from Scotland were serotyped as O86:K61, an isolate from Alaska (1297-05-019) did not react with any of the 175 O *E. coli* antiserum samples, including O86. Variable use of β-galactosidase and fermentation of d-sorbitol resulted in API 20E codes of 5144502 or 4144102 and more robust identifications as *E. coli* (84% and 90%, respectively).

The nearly full-length 16S rRNA sequence of isolate 1297-05-19 from the redpoll in Alaska was most similar (1,470 [99.7%] of 1,475 nt) to sequences of *E. albertii* (AY696669) and *S. boydii* (1,467 [99.5%] of 1,475 nt, AY696670) isolated from humans. The 495-nt sequence of the 5′ end of 16S rRNA was determined for redpoll 5419-05-R from Canada, finch EC37098 from Scotland, and gyrfalcon 12055-07 and chicken 7991-07 from the United States. In all, 9 nucleotide polymorphisms were observed among these 5 isolates, including 5 clustered in the V1 region (98.2% overall identity). These sequences were 99.2%–99.6% identical to the sequence of an *eae-*positive strain of *Hafnia alvei* (Z83203), *E. albertii* from humans (AJ508775, AY696662–AY696664, AY696669), and *S. boydii* serotypes 7 and 13 (AY696670–AY696680). PCRs were positive for the *E. albertii*–specific alleles of *lysP* and *mdh* (*10*) in all isolates from birds. Collectively, these data tentatively identified these isolates as *E. albertii*.

### Virulence Genes

**Figure 2:**
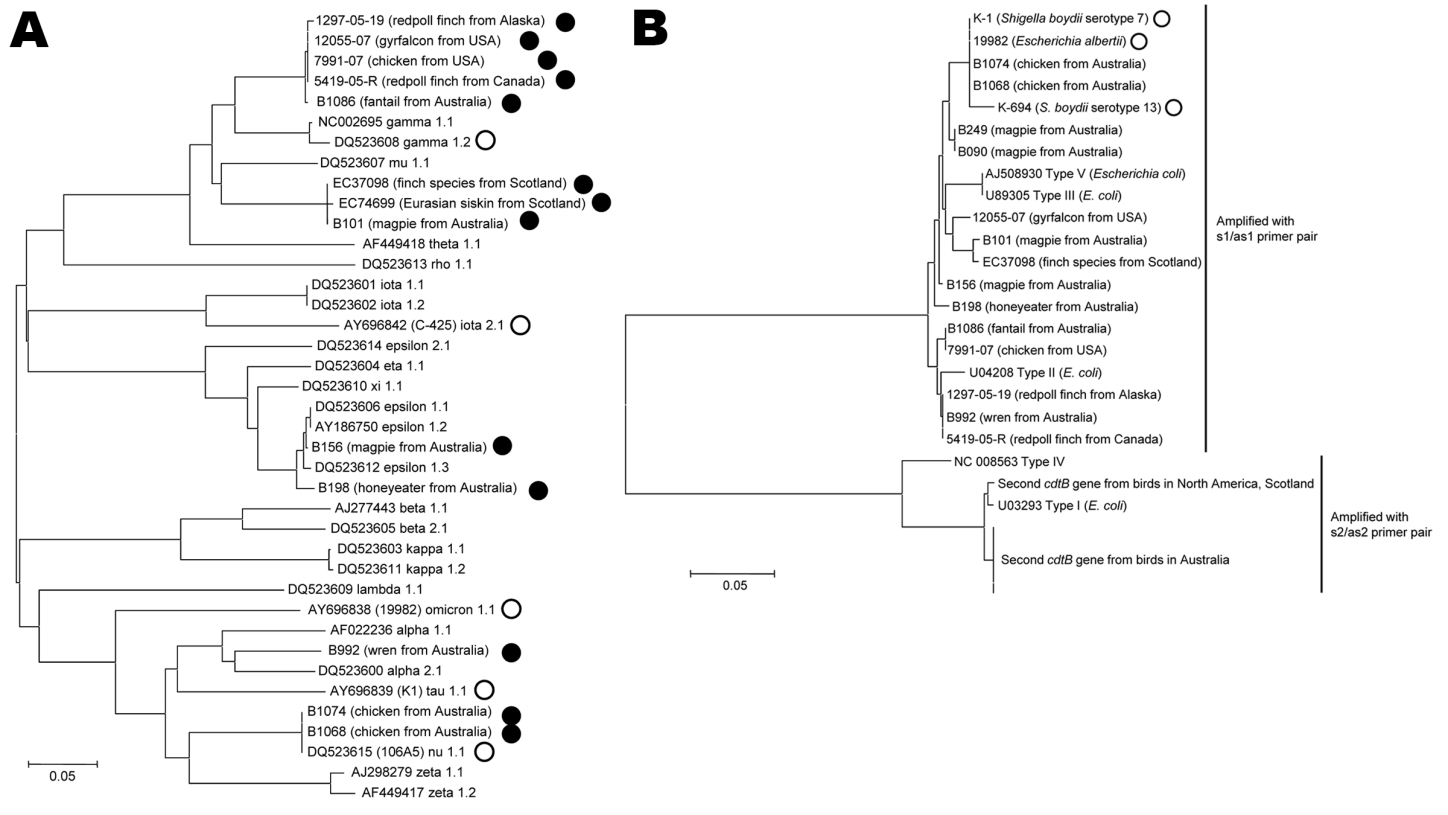
Neighbor-joining dendrogram based on the predicted amino acid sequences of the intimin (*eae*) and cytolethal distending toxin (*cdtB*) loci of *Escherichia* spp. Isolates analyzed in this study are designated by their isolate identification number; reference alleles are designated by their GenBank accession numbers. All *Escherichia albertii* isolates are indicated by circles or squares; all other isolates are *E. coli*. Scale bars indicate genetic distance. A) *eae* alleles carried by *E. albertii* isolates from birds (filled circles) represent diverse allelic subtypes and do not form separate clusters from allelic subtypes carried by isolates from humans (open circles). The *eae* alleles from North America and 1 from Australia (B1086) are novel but most similar to γ intimins. The *eae* alleles from Scotland and 1 from Australia (B101) are also novel but most similar to μ intimins. B) The *E. albertii cdtB* alleles from bird isolates, amplified by the s1/as1 primers, are most similar to *cdtB* types II, III, and V. *E. albertii* alleles from human isolates are marked with open circles. The bird *E. albertii cdtB* alleles amplified by the s2/as2 primers are distantly related to the other bird and human *E. albertii* alleles and are most similar to *cdtB* type I in *E. coli*. Because all the avian alleles amplified by the s2/as2 primers in each group are identical, only 1 sequence for each is shown.

All isolates from birds were positive for *eae* and *cdtB* but negative for *stx1*, *stx2*, and *sta*, the same repertoire of virulence genes reported for *E. albertii* isolates from humans (*10*). Alignment of the 3′ portion of the *eae* gene showed that the bird isolates possessed a variety of *eae* alleles, some novel and some similar to previously reported alleles (Figure 2, panel A). There was no clustering of bird *eae* alleles related to geographic origin, bird versus human origin, or isolation from diseased versus clinically healthy birds or humans. The largest cluster of bird alleles was found in representative isolates (Figure 2, panel A) from the redpolls from Alaska and Canada, the gyrfalcon and chicken from the United States, and the fantail from Australia, which were all nearly identical (1 nonsynonymous nt change each in the isolate from the redpoll and fantail from Alaska). This allele was distinct from other reference *eae* alleles and thus novel, but it was most similar to γ intimin. Alleles from the isolates from finch E37098 and siskin EC74699 from Scotland and magpie B101 from Australia were identical to each other but were also novel alleles most closely related to the μ allele in *E. coli* (*17*). The alleles from isolates from other wild birds and chickens from Australia were similar to previously reported allelic subtypes, including ε, α, and ν (*17*). Only the isolates from chickens B1068 and B1074 in Australia had an allelic subtype, ν 1.1, previously reported for an *E. albertii* isolate from a human (*10**,**17*).

All isolates tested (Figure 2, panel B) were PCR positive for *cdtB* with the s1/as1 primer pair. With the exception of the chicken from the United States and 5 isolates from birds in Australia (2 chickens [B1068 and B1074], 1 fantail, 1 honeyeater, and 1 wren), all were also positive for *cdtB* with the s2/as2 primer pair. Because these 2 primer pairs are specific for different types of cytolethal distending toxin (*16*), at least some isolates from birds appeared to carry multiple *cdtB* genes. Sequencing and alignment of the s1/as1 PCR products showed these to have ≈91% nt and 92% aa identity. On the basis of amino acid polymorphisms (Figure 2, panel B), avian *cdtB* alleles amplified by the s1/as1 primers were most similar to type II (birds from North America and Australia) and types III and V (the gyrfalcon, finches from Scotland, other birds from Australia) reference alleles (*21*–*23*). Sequencing and alignment of the s2/as2 PCR product showed that the sequences from the isolates from Australia were identical to each other, that the sequences from the isolates from North America and Scotland were identical to each other, and that these 2 groups of sequences were similar to each other with ≈99% identity at both the nucleotide and amino acid levels. These sequences were similar (1- or 2-aa differences) to the type I *cdtB* reference allele (*24*). The presence of a type I *cdtB* is consistent with the previous finding of a type I *cdtB* in the isolates from the finches from Scotland, identified by type-specific PCRs (*16*).

### MLST Findings

MLST of nucleotide variation at 6 loci (a total of 3,165 bp) in the genomes of isolates (Table 1) showed 3 main clades of *E. albertii* (EA 1, EA 2, and EA 3 in Figure 1). Isolates did not appear to cluster on the basis of host disease status (healthy, with diarrhea, or dead) or host type. All isolates from birds from North America were closely related and clustered in clade EA 2, along with 3 isolates from birds from Australia (honeyeater, wren, and fantail) and an isolate from a human with diarrhea (I2005002880 #36). The isolate from chicken 7991-07 was slightly divergent from the rest of the isolates from North America (5 synonymous and 1 nonsynonymous nt changes) and was indistinguishable from isolate I2005002880 #36 from the human. Isolates from the dead redpolls from Alaska, healthy finches from Canada, and the gyrfalcon were identical. The isolate from finch EC370-98 from Scotland was distantly related to other bird isolates and clustered with an isolate from a human with diarrhea (M2005000616 #8) in clade EA 1.

MLST strongly supported the biochemical and other molecular data indicating that the bird isolates in this study were *E. albertii*. Collectively, these isolates represent a distant relative of *E. coli*, a divergent lineage in the genus *Escherichia*, and novel diversity within the *E. albertii* species (Figure 1, inset).

### PFGE Findings

**Figure 3:**
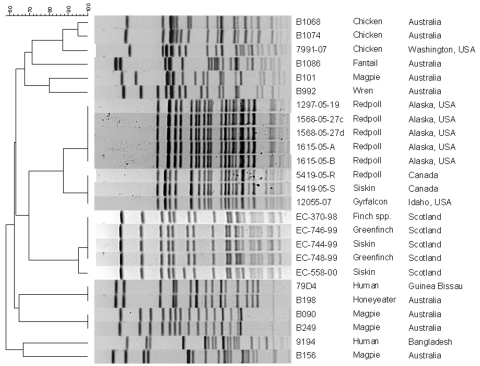
Dendrogram (unweighted pair group method with arithmetic mean) of *Escherichia albertii* isolates from birds, based on pulsed-field gel electrophoresis band profiles. Isolates from disease outbreaks in Alaska and Scotland form clonal groups, indicating that these outbreaks are associated with expansion of a single clone. The profiles for the other isolates indicate that the bird and human *E. albertii* isolates are heterogeneous and do not segregate on the basis of host, geographic origin, or disease status. Scale bar indicates percent similarity.

PFGE showed that isolates from the 2 bird death epornithics (in Alaska and Scotland) each formed a clonal group (Figure 3), which suggests that these events were associated with expansion of a single clone from either a common source or bird-to-bird transmission. Overall, the PFGE banding patterns and dendrogram indicate that the isolates from birds and humans constitute a heterogeneous group, consistent with the heterogeneity identified in *eae* and *cdtB* and by MLST.

## Discussion

*E. albertii* is a recently described member of the Enterobacteriaceae and has been associated with diarrheal illness in humans (*25*–*27*). Until now, however, it has not been associated with disease or infection in animals. *E. albertii* was originally described as an unusual strain of *H. alvei* with virulence genes that included *eae* and the *cdtABC* operon (*26*,*28*). Subsequent characterization of these *H. alvei* strains demonstrated that they were members of the genus *Escherichia* (*7*,*10*,*27*) and constituted a new taxon for which the name *E. albertii* was proposed (*7*). The *E. albertii* lineage diverged before the radiation of *E. coli* and *Shigella* spp. and includes the atypical *S. boydii* serotypes 7 and 13 (*10*). The prevalence, epidemiology, and clinical relevance of *E. albertii* are poorly defined, in part because *E. albertii* is likely to either remain unidentified or be misidentified by current commercial biochemical identification methods as *E. coli*, *H. alvei*, *S. boydii*, or *Yersinia ruckeri* (*7*,*29**,**30*).

Our phenotypic, biochemical, 16S rRNA sequence, and MLST analyses are in strong agreement that the bird isolates in this study, including the previously identified O86:K61 *E. coli* isolates from Scotland, are correctly classified as *E. albertii*. In addition, all bird isolates carried genes for 2 characteristic *E. albertii* virulence factors (intimin and cytolethal distending toxin). Our findings indicate that *E. albertii* is likely pathogenic to birds and can be associated with epornithics and sporadic disease. The primary pathologic lesion in birds was consistent with enteritis, but the classic attaching-and-effacing lesions typically associated with *eae*-positive pathogens were not detected. The postmortem condition of the dead finches may have prevented such detection, but experimental work with chicks and the isolates from Scotland suggests that other disease mechanisms need to be considered (*9*).

We also conclude that *E. albertii* is able to subclinically colonize various species of wild birds globally. The determinants of pathogenicity of *E. albertii* in birds remain to be clarified, but its isolation from diseased and healthy birds suggests that its epizoology in songbirds may resemble that of *S. enterica* subsp. *enterica* serotype Typhimurium, which is maintained by subclinical carriers and causes outbreaks of disease under conditions of increased stress or high bacterial doses (*5*,*6*,*31*).

The *E. albertii* isolates from birds in this study differed from those from humans in several notable ways, although the lack of phylogenetic clustering based on host of origin suggests that it would be premature to conclude that these differences are truly host related. First, all bird isolates produced indole from tryptophan, resulting in weak (43% level of confidence) identification as *E. coli* in contrast to indole-negative isolates from humans, which are identified as *H. alvei* (45% level of confidence) according to API 20E databases. However, when the positive indole result is combined with the positive reaction for d-sorbitol found for some bird isolates, the identification as *E. coli* is more robust (84%) and thus more likely to lead to misidentification. Second, we demonstrated the presence of 2 different *cdtB* genes. Whether the presence of multiple *cdtB* genes in the human or other bird *E. albertii* isolates was missed for technical reasons, or whether these other isolates contain a single gene, requires further investigation.

In conclusion, *E. albertii* appears to be a pathogen of animals and humans and may be carried subclinically by some birds. *E. albertii* is a member of a more heterogeneous group than was previously appreciated, and additional variation will likely become apparent as additional isolates from other animal hosts and geographic regions are characterized. Whether *E. albertii* can be transmitted from animals to humans is unknown, although the *eae*, *cdtB*, MLST, and PFGE data indicating that the bird isolates cluster among isolates from humans suggest that zoonoses or anthroponoses are possible. Regardless, identification of *E. albertii* in the clinical laboratory remains a challenge, and it is likely that this pathogen is often unidentified or misidentified in human and veterinary medicine.
